# Case report: the ‘vanished’ left pulmonary artery

**DOI:** 10.1093/ehjcr/ytae147

**Published:** 2024-03-28

**Authors:** Jiahui Charmaine Chan, Monika Kantilal Kotecha, Jonathan Tze Liang Choo, Marielle V Fortier, Sreekanthan Sundararaghavan

**Affiliations:** Department of Paediatric Subspecialties, Cardiology Services, KK Women and Children’s Hospital, 100 Bukit Timah Road, Singapore 229899, Singapore; Yong Loo Lin School of Medicine, National University of Singapore, 100 Medical Drive, Singapore 117597, Singapore; Duke-National University of Singapore Medical School, 8 College Rd, Singapore 169857, Singapore; Lee Kong Chian School of Medicine, Nanyang Technological University, 11 Mandalay Road, Singapore 308232, Singapore; Department of Paediatric Subspecialties, Cardiology Services, KK Women and Children’s Hospital, 100 Bukit Timah Road, Singapore 229899, Singapore; Yong Loo Lin School of Medicine, National University of Singapore, 100 Medical Drive, Singapore 117597, Singapore; Duke-National University of Singapore Medical School, 8 College Rd, Singapore 169857, Singapore; Lee Kong Chian School of Medicine, Nanyang Technological University, 11 Mandalay Road, Singapore 308232, Singapore; Department of Paediatric Subspecialties, Cardiology Services, KK Women and Children’s Hospital, 100 Bukit Timah Road, Singapore 229899, Singapore; Yong Loo Lin School of Medicine, National University of Singapore, 100 Medical Drive, Singapore 117597, Singapore; Duke-National University of Singapore Medical School, 8 College Rd, Singapore 169857, Singapore; Lee Kong Chian School of Medicine, Nanyang Technological University, 11 Mandalay Road, Singapore 308232, Singapore; Yong Loo Lin School of Medicine, National University of Singapore, 100 Medical Drive, Singapore 117597, Singapore; Duke-National University of Singapore Medical School, 8 College Rd, Singapore 169857, Singapore; Department of Diagnostic and Interventional Imaging, KK Women and Children’s Hospital, Singapore, Singapore; Singapore Institute for Clinical Sciences, Agency for Science, Technology and Research (A*STAR), Singapore, Singapore; Department of Paediatric Subspecialties, Cardiology Services, KK Women and Children’s Hospital, 100 Bukit Timah Road, Singapore 229899, Singapore; Yong Loo Lin School of Medicine, National University of Singapore, 100 Medical Drive, Singapore 117597, Singapore; Duke-National University of Singapore Medical School, 8 College Rd, Singapore 169857, Singapore; Lee Kong Chian School of Medicine, Nanyang Technological University, 11 Mandalay Road, Singapore 308232, Singapore

**Keywords:** Ductal origin of pulmonary artery, Discontinuous pulmonary arteries, Unifocalization, Pulmonary artery agenesis, Pulmonary artery stent, Bilateral ductus arteriosus, Case report

## Abstract

**Background:**

We report a case of isolated ductal origin of pulmonary artery (DOPA) diagnosed in an asymptomatic newborn. The primary aim of this case is to highlight the need to investigate for DOPA in patients diagnosed with an ‘absent branch pulmonary artery’.

**Case summary:**

Our patient was an asymptomatic newborn infant, with normal intracardiac anatomy. He was initially diagnosed post-natally with ‘absent left pulmonary artery’ (LPA), though the LPA was seen in antenatal scans. He underwent angiography and was re-diagnosed with bilateral arterial ducts, with ductal origin of the LPA from the left arterial duct. The LPA was salvaged by first stenting the left arterial duct on Day 11 of life, with subsequent surgery to connect the LPA to the main pulmonary artery at 4.5 months old. The patient had an uneventful recovery after the surgery.

**Discussion:**

Ductal origin of pulmonary artery is a rare vascular anomaly characterized by continuity of the left or right pulmonary artery (PA) with the distal end of the arterial duct, and discontinuity with the main PA. It is commonly misdiagnosed as pulmonary artery agenesis when the patent arterial duct constricts, with cessation of blood flow into the affected pulmonary artery. A high index of suspicion is necessary for diagnosis of DOPA. Once diagnosed, this lesion is clearly amenable to intervention, with benefits from unifocalization, to prevent late onset pulmonary hypertension or cardiac failure.

**Learning points**

- This case highlights the importance of investigating for ductal origin of pulmonary artery with angiography in patients diagnosed with absent branch pulmonary artery or pulmonary artery agenesis.
- Patients may be initially asymptomatic. If left untreated, however, they may develop pulmonary hypertension or cardiac failure.
- In neonates or young infants, a staged approach of stenting the ductus arteriosus prior to unifocalization may be favoured, allowing for physical growth of the baby, as well as the pulmonary artery, improving the surgical outcome.
- Antenatal diagnosis of the condition is possible, and should be suspected, if confluent branch pulmonary arteries cannot be demonstrated.

## Introduction

Ductal origin of pulmonary artery (DOPA) is a rare vascular anomaly characterized by continuity of a branch pulmonary artery (PA) with the distal end of the arterial duct, and discontinuity with the main PA. Its incidence is estimated at 1:200 000, however this may be underestimated as many cases are misdiagnosed as pulmonary artery agenesis or ‘absent pulmonary artery’.^1^ Untreated, long-term consequences include unilateral lung hypoplasia, pulmonary hypertension, and cardiac failure. Early diagnosis and repair have good prognosis.^2,3^

## Summary figure

**Table:** 

Antenatal	Pre-natal echocardiogram done at a private clinic at 22 + 6 weeks gestational age suggested double aortic arch
	Patient was transferred to our centre for further management at 32 weeks gestational age
Post-natal	
D2	Post-natal echocardiogram showed right aortic arch, absent left pulmonary artery (LPA), normal intracardiac anatomy
D9	CT angiography showed absent left pre-hilar LPA. Post-hilar left pulmonary arteries were seen. Possible bilateral arterial ducts (PDA) that had constricted
	High dose prostaglandin E2 initiated
D11	Cardiac catheterization study performed. Right aortic arch with bilateral PDA noted, discontinuous branch pulmonary arteries, with LPA arising from left PDA. Left PDA stented, re-establishing flow into LPA
	Complicated by left lung reperfusion injury, and thrombo-embolic stroke post-cardiac catheterization
D25	Discharged well from hospital
3 months old	Repeat CT angiography showed patent left PDA, good interval growth of post-hilar distal LPA
4.5 months old	Surgical re-connection of LPA to main pulmonary artery, and removal of left PDA stent
	Discharged home on post-operative Day 4

We report a case of an asymptomatic neonate with right aortic arch, bilateral arterial ducts, discontinuous pulmonary arteries with ductal origin of left pulmonary artery, who underwent successful staged repair, in a low volume cardiac centre.

## Case summary

**Figure 1:**
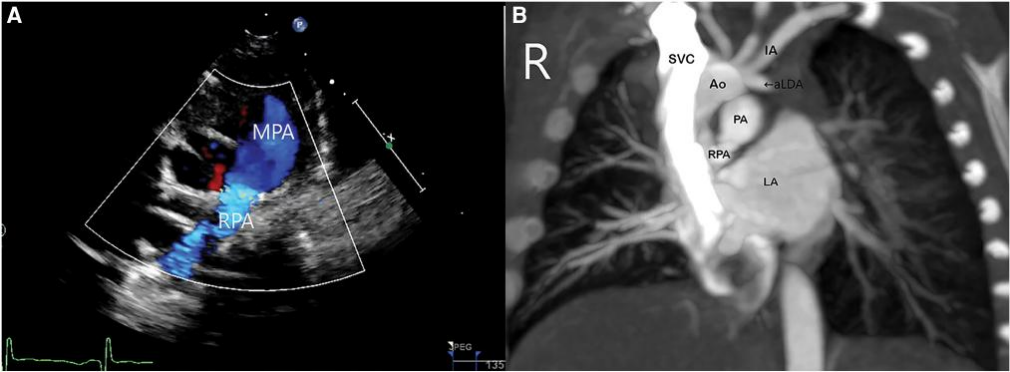
(*A*) Post-natal transthoracic echocardiography parasternal short axis view demonstrating flow from MPA to RPA. LPA was not visualized. (*B*) CT angiography demonstrating RPA arising from MPA. LPA again not visualized. A stump appearing from left IA can be seen, likely ampulla of left ductus arteriosus. MPA, main pulmonary artery; RPA, right pulmonary artery; SVC, superior vena cava; IA, innominate artery; Ao, aorta; aLDA, ampulla of left ductus arteriosus; PA, pulmonary artery; LA, left atrium.

A baby boy with an antenatal diagnosis of double aortic arch was born well at term, birth weight 3224 g. His saturations were ≥95% in room air. Post-natal echocardiography done on Day 2 of life showed normal intracardiac anatomy, right aortic arch, right pulmonary artery arising normally from main pulmonary artery (MPA), with left pulmonary artery (LPA) not visualized (*Figure 1A*).

Computer tomography (CT) angiography scan (*Figure 1B*) performed on Day 9 of life confirmed echocardiographic findings of absent left pre-hilar LPA. Post-hilar left upper and lower lobe pulmonary arteries were visualized. There were no major aortopulmonary collateral arteries. The ampulla of the right arterial duct was seen at the aortic isthmus. A short tubular vessel was seen arising from the left brachiocephalic artery, suspected to be the ampulla of a constricted left arterial duct (PDA). Both lungs were well developed.

**Figure 2:**
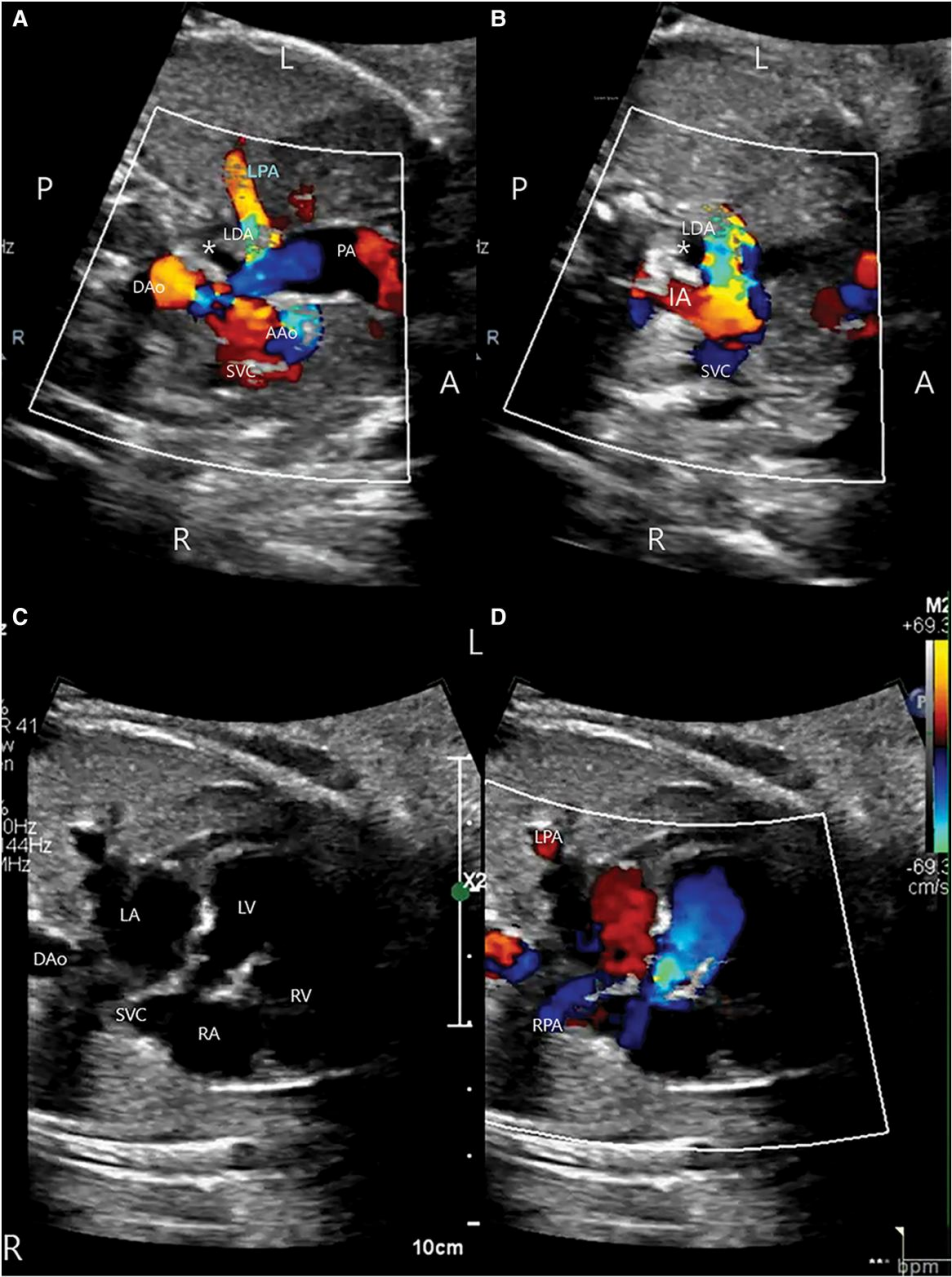
(*A* and *B*) Antenatal echocardiography at 32 weeks gestational age: a sweep depicting blood flow from PA to LDA to the isolated LPA. The LDA arises from the base of the left IA. (*C* and *D*) Sweep from four-chamber view, to three-vessel view, to the neck vessels, demonstrates that the LPA and RPA are discontinuous (see Supplementary material online, *Video S1A* and *B*).

On retrospective review of the antenatal echocardiogram performed at 32 weeks gestational age, we were able to trace the LPA from the lung parenchyma to the origin of left brachiocephalic artery (*Figure 2A*–*D*, Supplementary material online, *Video S1A* and *B*). We postulated that this patient had bilateral PDAs *in utero*, discontinuous branch pulmonary arteries, with the LPA being supplied by the left PDA. High dose prostaglandin E1 was commenced, however the PDA did not reopen. The patient remained asymptomatic, maintaining oxygen saturations ≥ 95% in room air.

**Figure 3:**
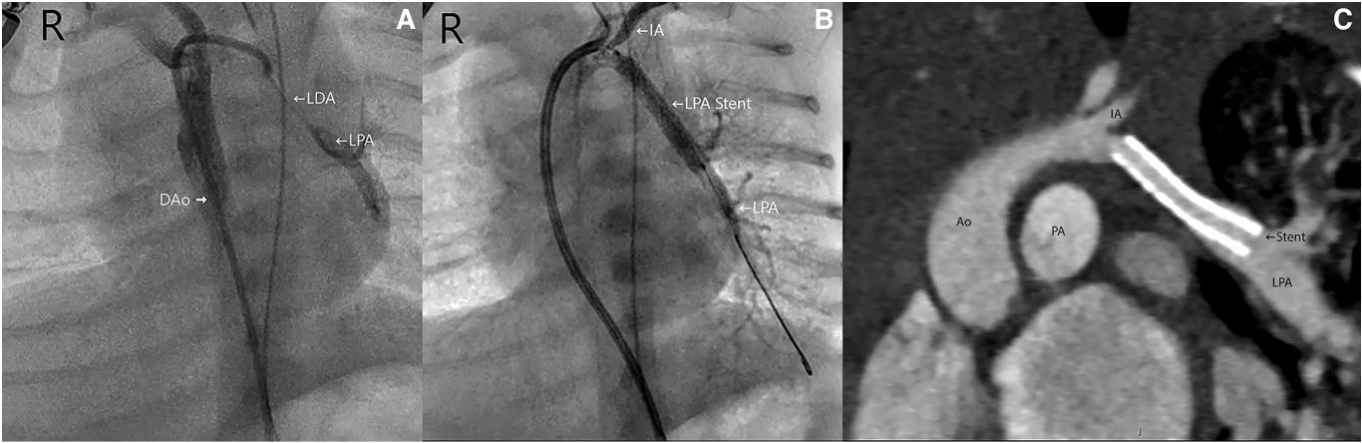
(*A*) Anteroposterior view of angiography showing thread-like LDA connecting from left innominate artery to LPA. (*B*) LPA stent re-establishes flow to distal LPA (see Supplementary material online, *Video S2A* and *B*). (*C*) CT angiography repeated 2 months post-stenting. The stent was patent, with good growth of distal LPA. DAo, descending aorta; LDA, left ductus arteriosus; LPA, left pulmonary artery; IA, innominate artery; Ao, aorta; PA, pulmonary artery.

Cardiac catheterization study was performed on Day 11 of life. Contrast injected at the origin of the left brachiocephalic artery demonstrated a thread-like connection with the LPA via the left PDA, confirming the diagnosis of ductal origin of the LPA. The left PDA was stented, with good pulmonary arterial blood flow established in all lobes of the left lung, and good pulmonary venous return (*Figure 3A* and *B*, Supplementary material online, *Videos S2* and *S3*).

Post-intervention, there was initial left lung reperfusion injury with pulmonary haemorrhage. This was medically managed with high positive end-expiratory pressure and diuresis. The patient also developed thrombo-embolic stroke presenting with focal seizures, for which he was treated with subcutaneous enoxaparin, titrated to maintain therapeutic anti-factor Xa levels (0.5–1.0 units/mL), along with oral phenobarbitone at 5 mg/kg/day. There was no neurological deficit clinically. However, magnetic resonance imaging (MRI) of the brain showed evidence of small areas of acute infarcts.

He was discharged on Day 25 of life. Good growth and saturations were noted on outpatient follow-up. Serial echocardiograms showed patent left PDA stent. The left atrium and ventricle were dilated, with mild mitral regurgitation. A repeat CT angiography at 3 months of age (*Figure 3C*) showed good interval growth of the post-hilar distal LPA. Repeat MRI brain showed mild gliosis from old infarcted areas, hence enoxaparin was discontinued while phenobarbitone was to be continued till re-assessment at 1 year of age.

**Figures 4:**
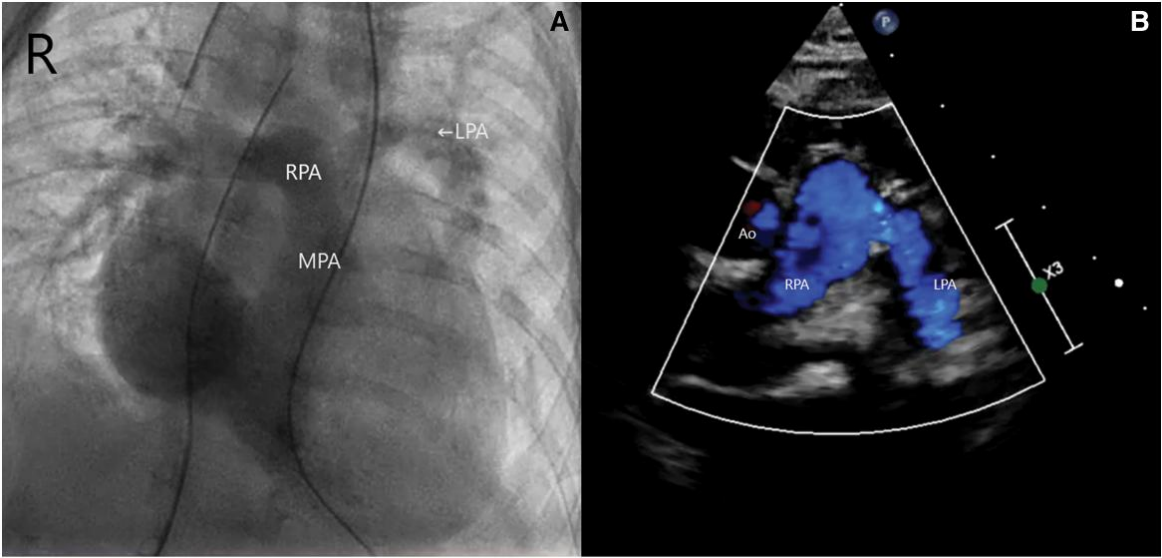
(*A* and *B*) Post-operative fluoroscopy demonstrates confluent branch pulmonary arteries. This was also demonstrated on echocardiography, with non-turbulent flow across both PAs seen. This is also demonstrated on fluoroscopy. RPA, right pulmonary artery; MPA, main pulmonary artery; LPA, left pulmonary artery; Ao, aorta.

At 4.5 months old, he underwent surgery to reconnect the LPA to MPA via direct anastomosis, using MPA flap to augment the anastomotic site, with removal of the left PDA stent. He was extubated hours after surgery uneventfully. Pre-discharge fluoroscopy and echocardiography (*Figure 4A* and *B*) demonstrated confluent and good-sized branch PAs, with non-turbulent flow across them. He was discharged well on post-operative Day 4.

He was last reviewed at 5.5 months of age in clinic. He remained well, with appropriate growth and development for age, and no further reported seizures. Echocardiography again demonstrated normal sized and non-turbulent branch pulmonary arteries. At 6 months of age, he and his family relocated back to their home country, and further follow-up was continued in their home country.

## Discussion

Ductal origin of pulmonary artery can occur with other congenital heart lesions, and less commonly, in isolation.^3^ Isolated DOPA may result in delayed diagnosis as it is frequently asymptomatic during childhood.

Embryologically, the distal intrapulmonary arteries arise from their respective lung buds, then join the proximal part of the sixth arch, forming the left and right pulmonary arteries. The distal portion of the sixth arches become the arterial duct. Regression of the proximal sixth arch, with persistence of its distal portion, results in ductal origin of the pulmonary artery.^4^ True pulmonary artery agenesis may occur if there is unilateral lung agenesis.

A high index of suspicion is required for the diagnosis, and should be considered in the absence of identifying confluent pulmonary arteries. CT angiography or MRI may be performed to determine if intrapulmonary arteries are present. A PDA or ligamentum arteriosus may also be identified on the ipsilateral side of the ‘absent’ PA.^3^ Angiography is the gold standard in diagnosis. Pulmonary venous wedge injections may depict hypoplastic intrapulmonary vessels.^5^

Pre-natal diagnosis of DOPA has been reported in a handful of cases.^6–13^ When confluent branch pulmonary arteries cannot be demonstrated in the three-vessels (3V) or three-vessels trachea view (3VT), a transverse sweep (3VT view) is performed from the level of the bifurcation of pulmonary arteries to the level above the aortic arch, demonstrating the head and neck vessels. Identification of the origin of the affected vessel arising from the base of the brachiocephalic artery via the ipsilateral patent arterial duct in this sweep clinches the diagnosis of DOPA.^13^

Factors contributing to our missed antenatal diagnosis included late presentation to our centre, suboptimal echocardiographic windows, and false reassurance from visualizing normal intraparenchymal pulmonary arteries, with normal intracardiac anatomy. The observation that the LPA had ‘vanished’ post-natally made us suspicious of DOPA, which was confirmed with angiography.

Management strategies involve either (1) direct unifocalization, or (2) staged procedure with either stenting of the arterial duct or placement of a surgical shunt. If the lesion was detected antenatally, prostaglandin E1 infusion may be immediately commenced post-natally to maintain ductal patency, before proceeding to either options.

Two large case series evaluating the outcomes of children with DOPA^2,3^ showed the benefit of intervention even in late presentation. Pulmonary blood flow was re-established in the affected lung, and none of the patients developed pulmonary hypertension during the follow-up period. The oldest reported patient in the study by Mery *et al*.^2^ was a child who underwent PDA stent at 9 years old, followed by unifocalization at 11 years old. In both case series, reinterventions (transcatheter or surgical) were required in the majority of patients to augment the growth of the reimplanted pulmonary artery, regardless of the initial approach chosen.

**Figure 5:**
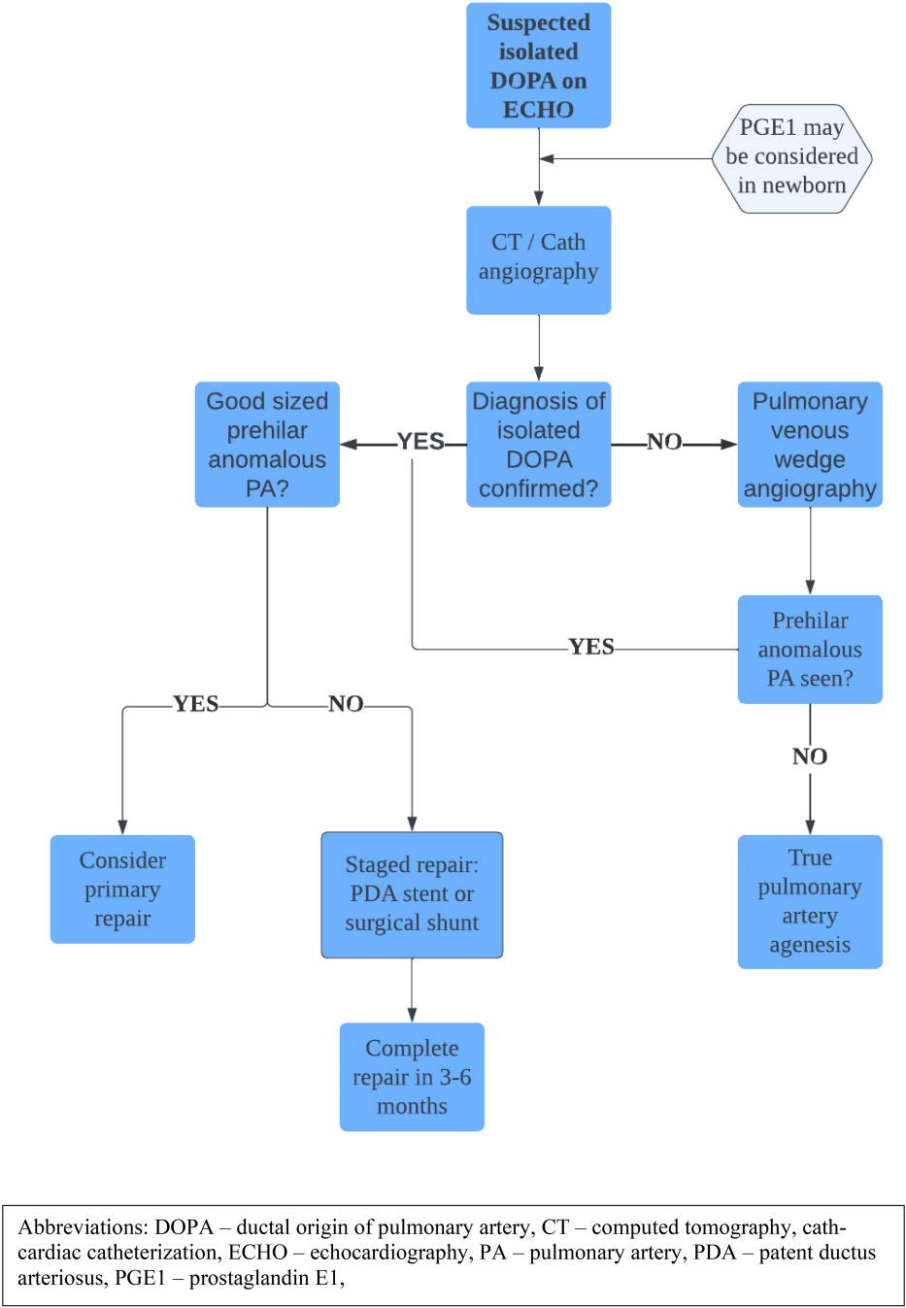
Flow chart: proposed management approach to isolated DOPA.

We chose a staged approach with ductal stenting followed by unifocalization for our patient, after discussion with our cardiothoracic surgeon. This approach allowed for good interim physical growth for our patient, as well as distal LPA growth, reducing the technical challenges of surgery, and the patient had a quick post-operative recovery. A recent multicentre review^14^ found that in children with isolated pulmonary artery of ductal origin (IPADO), with or without structural heart defects, a staged repair was associated with larger isolated PA size and symmetry at late follow-up, as compared to primary unifocalization. We have proposed a management approach to isolated DOPA (*Figure 5*).

## Conclusion

In conclusion, isolated DOPA is a rare condition that should be considered when the diagnosis of ‘absent pulmonary artery’ is made. It can be diagnosed antenatally if the affected pulmonary artery is demonstrated to arise from the ipsilateral brachiocephalic artery. Reconnecting the isolated PA to MPA should always be attempted, which reduces the risk of long-term consequences such as pulmonary hypertension and cardiac failure. A staged procedure may improve the surgical risk profile in young infants in a low volume cardiac unit.

## Supplementary Material

ytae147_Supplementary_Data

## Data Availability

The authors confirm that all data supporting the findings of this case report are available within the manuscript and its online supplementary material.

## Lead author biography

Charmaine Chan is a consultant in the Department of Paediatric Cardiology, KK Women and Children’s Hospital, Singapore. She graduated from University of Otago, Dunedin, New Zealand, and subsequently completed her Paediatric residency and Paediatric Cardiology residency in Singapore. She is interested in congenital heart diseases and paediatric electrophysiology.
